# Efficacy of acupuncture combined with traditional Chinese medicine on chronic prostatitis: A protocol for systematic review and meta-analysis

**DOI:** 10.1097/MD.0000000000027678

**Published:** 2021-11-19

**Authors:** Xianglong Zheng, Zhangren Yan, Wanchun Wang, Wenli Mao, Yuhan Wang, Yanling Zhao, Zhiying Zhong

**Affiliations:** a Jiangxi University of Chinese Medicine, Nanchang, China; b The Affiliated Hospital of Jiangxi University of Chinese Medicine, Nanchang, China.

**Keywords:** acupuncture, chronic prostatitis, meta-analysis, protocol, systematic review, traditional Chinese medicine

## Abstract

**Background:**

Chronic prostatitis is a common andrological disease, which brings many troubles to the lives of middle-aged and elderly male patients. With the increase of modern life pressure, the incidence of chronic prostatitis tends to younger, but its etiology and pathogenesis are not fully elucidated. Which seriously affects men's health? Relevant studies have shown that acupuncture combined with traditional Chinese medicine (TCM) has a good effect on the treatment of chronic prostatitis compared with conventional western medicine; however, there is no consistent conclusion at present. The main purpose of this study is to explore whether acupuncture combined with TCM is effective in treating chronic prostatitis.

**Methods:**

The collection of randomized controlled trials related to acupuncture and TCM for chronic prostatitis will search the following electronic databases, including: PubMed, Web of Science, the Cochrane Database, EMBASE, Chinese National Knowledge Infrastructure, Wanfang Data Knowledge Service Platform, Weipu. There are 8 electronic databases including the VIP Chinese Science and Technology Periodical Database and the China Biomedical Literature Database. The cure rate and total effective rate are the main indicators, and the recurrence rate and adverse events are the secondary indicators. Meta-analysis using RevMan5.4 provided by Cochrane Collaboration.

**Results:**

This study will provide the latest evidence of efficacy for the acupuncture combined with TCM in the treatment of chronic prostatitis.

**Conclusion:**

The effectiveness of acupuncture combined with TCM for chronic prostatitis will be evaluated.

**Unique INPLASY number:**

INPLASY202130083.

## 1 Introduction

### 1.1 Objective

This study comprehensively searched the literature to further systematically evaluate the efficacy of acupuncture combined with traditional Chinese medicine (TCM) in the treatment of chronic prostatitis,^[1]^ to clinically treat chronic prostatitis, alleviating its related clinical symptoms and preventing its further development, and providing the latest evidence-based medical evidence.^[2]^

### 1.2 Condition being studied

Chronic prostatitis is a urinary tract disease with the main clinical manifestations of lower abdominal pain and abnormal urination symptoms such as frequency, urgency, and insufficiency of urination.^[3,4]^ The incidence of chronic prostatitis in men under the age of 50 is up to 8% worldwide.^[5,6]^ In addition, The recurrence of chronic prostatitis will affect the quality of work and life of modern men to varying degrees.^[7]^ The exact pathogenesis of this disease is complex and has not been illustrated completely.^[8]^ Currently, the main treatment methods focus on autoimmunity, pathogens, mast cells, inflammatory factors, and other pathogenic factors.^[9]^ At present, the western medicine treatment of chronic prostatitis is mainly the combined application of antibiotics, anti-inflammatory drugs, α-adrenergic receptors, PDE-5 inhibitors and other drugs, including shockwave therapy, which is gradually used in clinical practice, mainly to improve symptoms.^[10,11]^ Although there are various treatment schemes, the efficiency is not high, with potential side effects.^[9]^

Nonetheless, an increasing number of studies have confirmed that acupuncture combined with the internal administration of TCM is effective in the treatment of chronic prostatitis.^[4,12,13]^ And compared with western medicine, acupuncture has no side effects and drug dependence, and the effect is significant.^[14]^ In the process of acupuncture treatment, umbilical needle can be used to regulate the activities of Zang-Fu organs, promote qi and blood circulation, and relieve pain. It has the advantages of multiple treatment at one point, multiple acupuncture at 1 point, multiple effect at 1 point, and both internal and external treatment.^[15]^ Acupuncture can reduce the levels of proinflammatory factor, increase anti-inflammatory factor level, regulate inflammatory factor levels, thus plays the role of immune regulation. can improve the body's ability to resist oxidative stress, regulate the body's oxidation and antioxidant balance, to alleviate the tissue damage, can adjust the neurotransmitter and autonomic nerve adjust nerve function, and by adjusting the pelvic floor muscle contraction to improve urine dynamics, relieve the symptoms of abnormal urination.^[16]^

The combination of internal and external treatment of TCM, oral prescription of TCM combined with TCM retention enema can promote the absorption of effective components in drugs, improve the utilization rate of drugs, enhance the curative effect, and have more therapeutic advantages.^[17]^ Although there have been randomized controlled trials (RCTs) that have shown acupuncture to be effective, but systematic evaluation research is still lacking.^[2]^

In order to preferably serve clinical decision making, this study will comprehensively search evidences and conduct a meta-analysis to the efficacy of acupuncture combined with TCM for chronic prostatitis.^[1]^ The implementation of this protocol will hope to alleviate clinical symptoms, prevent further development and recurrence, save health costs, and provide the latest evidence-based medical evidence.^[2]^

### 1.3 Protocol registration

This protocol will be conducted and reported by the guideline of the Preferred Reporting Items for Systematic Reviews and Meta-Analysis Protocols (PRISMA-P) and the PRISMA Extension Statement.^[18]^ This study protocol has been registered by us on the International Platform of Registered Systematic Review and Meta-analysis Protocols website (registration number:INPLASY202130083; https://inplasy.com/inplasy-2021-3-0083/).

## 2 Methods

### 2.1 Inclusion criteria for research selection

#### 2.1.1 Type of participants

All patients included in the trial were men under 50 years of age with chronic prostatitis with frequent urination ^[10,11]^, urgent urination, insufficiency, or periprostatic tissue pain, and B-ultrasonography showing calcification of the prostate, without race or territory.

#### 2.1.2 Type of interventions

The treatment group was mainly acupuncture combined with TCM. The control group consisted of patients receiving conventional western medicine treatment or any other intervention except acupuncture and TCM treatment.

#### 2.1.3 Type of comparators

The control group could receive conventional drug treatment or treatment other than acupuncture and TCM, or receive health education and counseling (ie, comfort treatment).

#### 2.1.4 Types of studies

A RCTs study published in any language of acupuncture combined with TCM in the treatment of chronic prostatitis will be included.

### 2.2 Exclusion criteria

(1) Non-RCT or RCT protocols literature.
(2) Animal trials, case reports and reviews, etc.
(3) Repeatedly detected or published literature.
(4) Unable to obtain complete data or full text literature.

### 2.3 Main outcomes and additional outcomes

#### 2.3.1 Main outcomes

Abnormal urination symptoms such as frequent urination, urgent urination, and insufficient urination disappeared; pain and distension in the scrotum, testicles, lower abdomen, and perineum disappeared; meanwhile, chronic prostatitis score index (National Institutes of Health Chronic Prostatitis Symptom Index) was referred to.

#### 2.3.2 Additional outcomes

B-ultrasonography showed the disappearance of prostate calcification, no white blood cells in prostatic fluid routine, and even distribution of lecithin bodies, no less than 3 “+”.^[14–16]^

### 2.4 Search strategy

PubMed, Web of Science, The Cochrane Database, Embase, Chinese National Knowledge Infrastructure, Wanfang Data Knowledge Service Platform, VIP Chinese Science and Technology Journal Database, China Biomedical Literature Database, and other 8 electronic databases. The retrieval time is from the establishment date to March 1, 2021. Search using keyword search. Chinese databases search terms include chronic prostatitis, acupuncture, TCM, random. English search terms include “ chronic prostatitis”, “ traditional Chinese medicine”, “ Chinese medicine” and “acupuncture”. This study does not limit the scope of language retrieval. Besides, we manually searched other literature, as well as unpublished research and conference materials. If the test report data is unknown or lacking, we will contact the author by email. The detailed search strategy of PubMed is shown in Table 1.

**Table 1:** Search strategy used in PubMed database.

Order	Search items
#1	Search “chronic prostatitis”[Mesh] OR“prostatitis” [Mesh] OR “chronic pelvic pain syndrome”[Mesh] Sort by: Publication Date
#2	Search (((chronic prostatitis [Title/Abstract]) OR prostatitis [Title/Abstract]) OR chronic pelvic pain syndrome [Title/Abstract]) Sort by: Publication Date
#3	#1 OR #2
#4	Search (((((((randomized controlled trial [Publication Type]) OR controlled clinical trial [Publication Type]) OR randomized [Title/Abstract]) OR drug therapy [MeSH Subheading]) OR placebo [Title/Abstract]) OR randomly [Title/Abstract]) OR trial [Title/Abstract]) OR groups [Title/Abstract] Sort by: Publication Date
#5	Search “traditional Chinese medicine” [MeSH] OR “acupuncture” [MeSH T] Sort by: Publication Date
#6	#4 AND #5
#7	#3 AND #6

### 2.5 Study selection and data extraction

Two review authors will independently assess the titles and abstracts of the search strategy's results in terms of their relevance and design and then inspect the full text of all potentially eligible studies according to the eligibility criteria.

**Figure 1:**
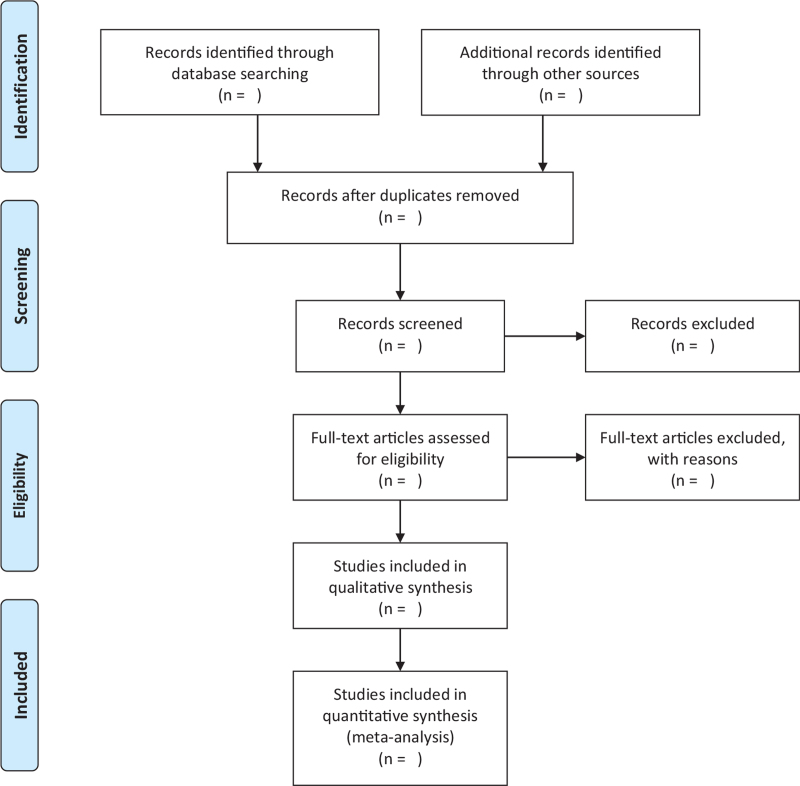
Flowchart of literature selection.

We will establish the document information extraction table in predesigned Excel. Two review authors will independently extract the following information from each included study: demographic characteristics of the subjects, sample size, treatment method, allocated to intervention and control groups, course of treatment, adverse events, data analysis strategy, effective rate, and outcome indicators. We will record the reasons for the excluded studies. The results extracted by 2 review authors will be cross-checked. Disagreements will be resolved by discussion and, where required, the input of a third review author. The specific process of study selection will be shown in the flow chart of PRISMA (Fig. 1).

### 2.6 Risk assessment of bias and quality assessment

Two review authors will independently use Cochrane Risk of Bias tool to appraise the risk of bias of each included study. Any discrepancy between 2 reviewers will be resolved by discussion and a third reviewer where necessary.

## 3 Analyse

### 3.1 Strategy of data synthesis

Our data uses RevMan5.4 provided by the Cochrane Collaboration for meta-analysis. Relative risk was used for 2 categorical variables, and mean difference was used for continuous variables. Both are expressed with 95% confidence interval. The test for heterogeneity among the results of the included studies used the *I*^*2*^ test. The *I*^*2*^ value reflects the proportion of the total change in the effect size due to the existence of heterogeneity. *I*^*2*^ > 50%, the heterogeneity is more obvious. If there is no obvious heterogeneity between the research results (*I*^*2*^ < 50%), the fixed effects model is used to merge; if there is significant heterogeneity (*I*^*2*^ > 50%), First analyze the source of heterogeneity, which may lead to heterogeneity factors in the subgroup analysis. If there is statistical heterogeneity in each subgroup and there is no clinical heterogeneity, the random effects model is used for analysis. If the heterogeneity is too large and the results cannot be combined, a descriptive analysis is used, and sensitivity analysis is performed if necessary.

### 3.2 Subgroup analysis

The subgroup analysis will be based on the method of acupuncture, different TCM decoctions, differences in patient conditions, and control groups.

### 3.3 Sensibility analysis

We will conduct a sensitivity analysis to verify the robustness of the review conclusions. The study design, methodological quality, and the impact of missing data will be evaluated. Sensitivity analysis is considered a study with low risk of bias.

### 3.4 Ethics and dissemination

All data in this study are from published studies and do not involve patients or the public, so ethical approval is not required. The results will be submitted for publication in a peer-reviewed journal.

## 4 Discussion

Chronic prostatitis can lead to seminal vesiculitis, affect the quality of sperm, cause semen nonliquefaction disease, which make them feel discomfort and pain, and even seriously affect your physical and mental health. Meanwhile, chronic prostatitis is an important cause of infertility, bringing about patients more serious mental pressure than the disease itself.^[19]^ Therefore, it is urgent to seek a thorough and effective treatment.^[20]^

Acupuncture combined with TCM therapy mainly treats clinical symptoms by regulating the function of bladder gasification. However, there is no clear conclusion yet. We will strictly follow the “Cochrane Manual Intervention Measures System Review”, conduct a systematic review and meta-analysis on the basis of existing RCTs, and evaluate the efficacy of acupuncture combined with TCM in the treatment of chronic prostatitis, mainly to provide a basis for clinical practice and future research.^[21]^

Chronic prostatitis has a high incidence and complex symptoms, which can easily cause negative pessimism in patients and increase the difficulty of treatment. Finding the best treatment method is an important task for our medical workers; the use of TCM combined with acupuncture to treat patients with this disease is easy to accept, has accurate curative effects, and has low side effects, and is worthy of promotion.

## Author contributions

**Conceptualization:** Xianglong Zheng, Wanchun Wang.

**Data curation:** Zhangren Yan, Yuhan Wang, Yanling Zhao.

**Formal analysis:** Wenli Mao.

**Methodology:** Zhangren Yan, Wenli Mao.

**Software:** Zhangren Yan, Zhiying Zhong.

**Supervision:** Xianglong Zheng, Wanchun Wang.

**Writing – original draft:** Xianglong Zheng, Yuhan Wang, Yanling Zhao.

**Writing – review & editing:** Xianglong Zheng, Yuhan Wang, Yanling Zhao.
